# Extremely thermophilic microorganisms as metabolic engineering platforms for production of fuels and industrial chemicals

**DOI:** 10.3389/fmicb.2015.01209

**Published:** 2015-11-05

**Authors:** Benjamin M. Zeldes, Matthew W. Keller, Andrew J. Loder, Christopher T. Straub, Michael W. W. Adams, Robert M. Kelly

**Affiliations:** ^1^Department of Chemical and Biomolecular Engineering, North Carolina State UniversityRaleigh, NC, USA; ^2^Department of Biochemistry and Molecular Biology, University of GeorgiaAthens, GA, USA

**Keywords:** extreme thermophiles, metabolic engineering, bio-based chemicals, genetics, biotechnology

## Abstract

Enzymes from extremely thermophilic microorganisms have been of technological interest for some time because of their ability to catalyze reactions of industrial significance at elevated temperatures. Thermophilic enzymes are now routinely produced in recombinant mesophilic hosts for use as discrete biocatalysts. Genome and metagenome sequence data for extreme thermophiles provide useful information for putative biocatalysts for a wide range of biotransformations, albeit involving at most a few enzymatic steps. However, in the past several years, unprecedented progress has been made in establishing molecular genetics tools for extreme thermophiles to the point that the use of these microorganisms as metabolic engineering platforms has become possible. While in its early days, complex metabolic pathways have been altered or engineered into recombinant extreme thermophiles, such that the production of fuels and chemicals at elevated temperatures has become possible. Not only does this expand the thermal range for industrial biotechnology, it also potentially provides biodiverse options for specific biotransformations unique to these microorganisms. The list of extreme thermophiles growing optimally between 70 and 100°C with genetic toolkits currently available includes archaea and bacteria, aerobes and anaerobes, coming from genera such as *Caldicellulosiruptor, Sulfolobus, Thermotoga, Thermococcus*, and *Pyrococcus*. These organisms exhibit unusual and potentially useful native metabolic capabilities, including cellulose degradation, metal solubilization, and RuBisCO-free carbon fixation. Those looking to design a thermal bioprocess now have a host of potential candidates to choose from, each with its own advantages and challenges that will influence its appropriateness for specific applications. Here, the issues and opportunities for extremely thermophilic metabolic engineering platforms are considered with an eye toward potential technological advantages for high temperature industrial biotechnology.

## Introduction

Microorganisms have been utilized for millennia in the production of food and beverages. With the advent of the industrial revolution, microbes were used to produce ethanol for fuel (Songstad et al., 2009), then acetone and butanol as chemical feedstocks during World War I (Jones and Woods, 1986). However, the discovery of easily accessible petroleum deposits, coupled with improvements in oil refineries, placed the biological routes at an insurmountable disadvantage for decades. The rising economic and environmental costs of petroleum-based products have renewed interest in biological production of commodity fuels, as well as specialty chemicals not easily synthesized via petrochemical routes. Most research in this area has focused on microbes growing in the mesophilic temperature range (25–37°C). However, high temperature fermentations, closer to the temperatures used in chemical refineries, are possible through the use of extremely thermophilic (T_opt_ ≥ 70°C) microbial hosts, offering a number potential of advantages over mesophilic biorefineries.

The enzymes of extreme thermophiles have been of considerable interest in biotechnology ever since the development of the polymerase chain reaction (Bartlett and Stirling, 2003). Given the usefulness of thermostable enzymes in molecular biological laboratory methods, it is not surprising that they have been proposed as powerful tools for industrial catalysis as well (Zamost et al., 1991; Vieille and Zeikus, 2001; Atomi et al., 2011). It is also becoming increasingly possible to improve the thermostability of mesophilic enzymes, either through protein engineering or techniques such as enzyme immobilization (Lehmann and Wyss, 2001; Harris et al., 2010; Steiner and Schwab, 2012; Singh et al., 2013), so that thermally-based bioprocessing can be considered.

High-temperature bioprocessing has a number of advantages, including reduced risk of contamination as compared to mesophilic hosts such as *E. coli* and *S. cerevisiae*, lowered chances of phage infection, improved solubility of substrates such as lignocellulosic biomass, continuous recovery of volatile chemical products directly from fermentation broth, and reduced cooling costs due to the greater temperature differential between the fermenter and the ambient air, which is the ultimate heat acceptor (Frock and Kelly, 2012; Keller et al., 2014). Increasing temperature can also make reactions that would be unfavorable in mesophiles thermodynamically feasible. In the industrial production of fructose from corn syrup via glucose isomerase, higher temperatures favor the fructose side of the reaction, creating a final product with better sweetening power (Bhosale et al., 1996). Hydrogen production becomes more favorable at high temperatures, leading to increased hydrogen productivities in thermophiles (Verhaart et al., 2010), and bioleaching of highly refractory ores such as chalcopyrite is more favorable under thermophilic conditions (Du Plessis et al., 2007). Methane production actually yields less energy at high temperature (Amend and Shock, 2001), but this leads to improved methane production in thermophilic methanogens (Ahring, 1995) since more methane must be generated to provide the same amount of cellular energy.

**Figure 1:**
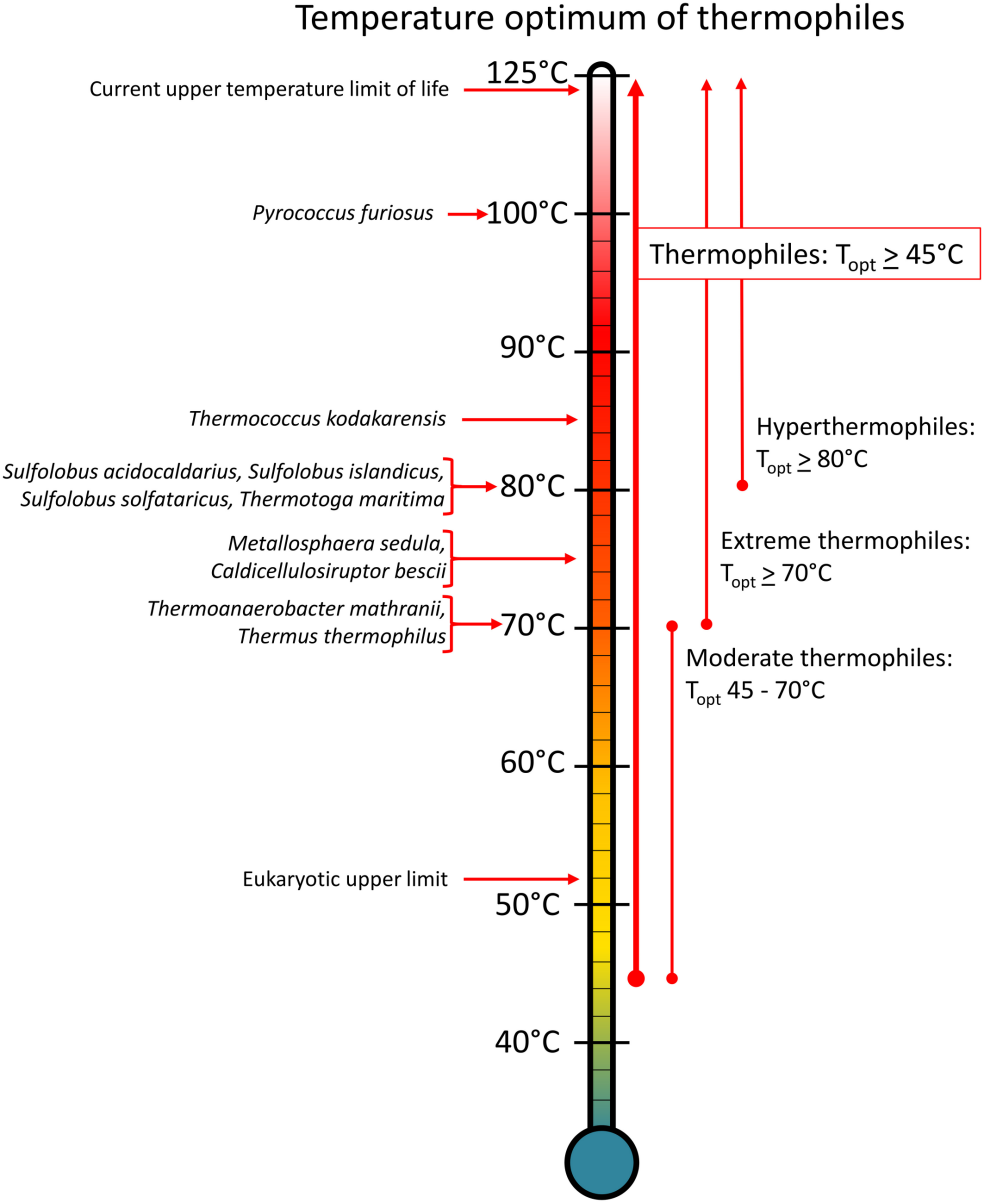
**Genetically tractable extreme thermophiles and their optimum growth temperatures**. Any organism with an optimum temperature above 45°C is classified as a thermophile, but this range is extremely broad and extends 80° units up to the upper limit of life. Therefore, thermophiles have been further subdivided into moderate thermophiles that grow optimally between 45 and 70°C, extreme thermophiles that grow optimally at 70°C and above, and hyperthermophiles that grow optimally at 80°C and above.

Thermostable enzymes can be used for *in vitro* single-step reactions, such as hydrolyzing large biopolymers into smaller components by proteinases, chitinases, cellulases, and other carbohydrate-degrading enzymes (Vieille and Zeikus, 2001). However, more complex multistep chemical conversions require an intact cellular host (Ladkau et al., 2014). Many interesting and potentially industrially relevant pathways require multiple enzyme steps, regeneration of cofactors, energy conservation via coupling to a transmembrane gradient, or input of additional chemical energy. The potential for extreme thermophiles to serve as intact platforms for metabolic engineering and whole-cell biocatalysts has been considered (Taylor et al., 2011; Frock and Kelly, 2012), but the field remains in its infancy (Figure 1).

## Genetics in extreme thermophiles

A major challenge to genetic modification in extreme thermophiles is establishing selective pressure for obtaining positive transformants. The antibiotics typically used in mesophiles often target cell components specific to bacteria, and so are ineffective against the archaeal species that dominate at high temperatures. Even in cases where antibiotics are effective, both the antimicrobial compound and the gene product that confers resistance to it must be stable at elevated temperatures. Because of the challenges with antibiotics, nutritional selection techniques such as those initially established in yeast genetics (Romanos et al., 1992), predominate in the genetic systems currently available for extreme thermophiles. Selective markers that have been successfully used in extreme thermophiles are summarized in Table 1.

**Table 1:** **Selective pressures for genetic manipulations in extreme thermophiles**.

	**Selection**	**Notes**
Nutrient-based	Uracil prototrophy	Effective in almost all cases, acceptor strain easily generated by growth on 5-FOA. Can suffer from high background due to contaminating uracil
	Tryptophan prototrophy	Less background than uracil, another selection mechanism required to generate acceptor strain, but can be used in conjunction with uracil prototrophic selection
	Agmatine prototrophy	Less background than uracil, another selection mechanism required to generate acceptor strain, but can be used in conjunction with uracil prototrophic selection
	Lactose utilization	Only effective for species capable of growth on lactose minimal media. Slow due to nutrient limitations
Antibiotics	Kanamycin	Bacteria specific
	Bleomycin	Bacteria specific
	Hygromycin	Bacteria specific
	Simvastatin	Archaea specific
Counter selection	5-Fluoroorotic acid	pyrF counterselection, requires uracil. Can also be used to generate the initial acceptor strain
	6-Methyl purine	Analogous to 5-FOA: counterselection requires adenine.

The most frequently used nutritional selection method in extreme thermophiles is based on uracil prototrophic selection from an auxotrophic parental strain. Synthesis of uracil involves enzymes encoded by the *pyrE* (orotate phosphoribosyltransferase) and *pyrF* (orotidine-5′-phosphate decarboxylase) genes that, besides their role in uracil production, also convert the synthetic chemical 5-fluoroorotic acid (5-FOA) into the cytotoxic fluorodeoxyuridine (Jund and Lacroute, 1970; Krooth et al., 1979). Therefore, growth of strains with functional uracil pathways on media containing 5-FOA selects for natural mutants with disruptions in *pyrE* or *pyrF* (Krooth et al., 1979; Worsham and Goldman, 1988).

While nutritional selection currently dominates the genetics of thermophiles, antibiotics have played a critical role in the development of these genetic systems. Simvastatin represents an important exception to the rule that antibiotics are only effective against mesophilic bacteria. It affects thermophilic archaea because it targets the production of archaeal membranes (Lam and Doolittle, 1989; Matsumi et al., 2007), and displays no detectable degradation at 100°C in the absence of oxygen (Simões et al., 2014). These important characteristics have allowed simvastatin to play a major role in the development of genetic systems in the hyperthermophilic archaea *Thermococcus kodakarensis* (Matsumi et al., 2007) and *Pyrococcus furiosus* (Waege et al., 2010).

Several other antibiotics are stable at high temperatures for durations adequate to provide selective pressure. Resistance genes that confer protection from these antibiotics at lower temperature can be evolved into more thermostable mutants for use at high temperature. This strategy has been enacted by cloning mutagenized kanamycin nucleotidyltryansferase into the thermophilic bacterium *Bacillus stearothermophilus*. The result is an increase in the temperature limit of kanamycin resistance from 47°C up to 70°C, which is near the 72°C limit of the antibiotic itself (Matsumura and Aiba, 1985; Liao et al., 1986). These thermostable mutants were further evolved to increase activity and thermostability to facilitate use in the thermophile *Thermus* (*Ts*) *thermophilus* (Hoseki et al., 1999). Bleomycin, a more thermostable antibiotic, was used to develop a hyperthermophilic (T_opt_ > 80°C) selectable marker; the engineered bleomycin-binding protein denatures at 85°C in the absence of bleomycin and 100°C in its presence (Brouns et al., 2005). Adding to the growing list of thermophilic selectable markers, *E. coli* hygromycin B phospotransferase was evolved for use in *Ts. thermophilus* at a maximum temperature of 67°C (Nakamura et al., 2005).

Natural competence—the ability to uptake and incorporate foreign DNA without exogenously applied pressure (such as divalent cations or an electric field)—is prevalent among extreme thermophiles, and has been proposed to play an important role in adaptation to extreme temperature (Averhoff and Müller, 2010). Some of the simplest and most frequently utilized genetic systems in the extreme thermophiles are in the species that exhibit natural competence: *Ts. thermophilus* (Koyama et al., 1986), *P. furiosus* strain COM1 (Lipscomb et al., 2011), and *T. kodakarensis* (Hileman and Santangelo, 2012). The benefit of natural competence is the simplicity of the transformation protocol, which involves simply mixing DNA with a dilute culture prior to selective plating. Transformation in other species is considerably more complex. Even though electroporation may be effective for introducing DNA constructs into the cell, once there, cellular defenses designed to destroy foreign DNA are problematic. Restriction enzymes in *C. bescii* degrade DNA that is not properly methylated, creating an added challenge for genetic manipulations in this organism (Chung et al., 2012).

**Figure 2:**
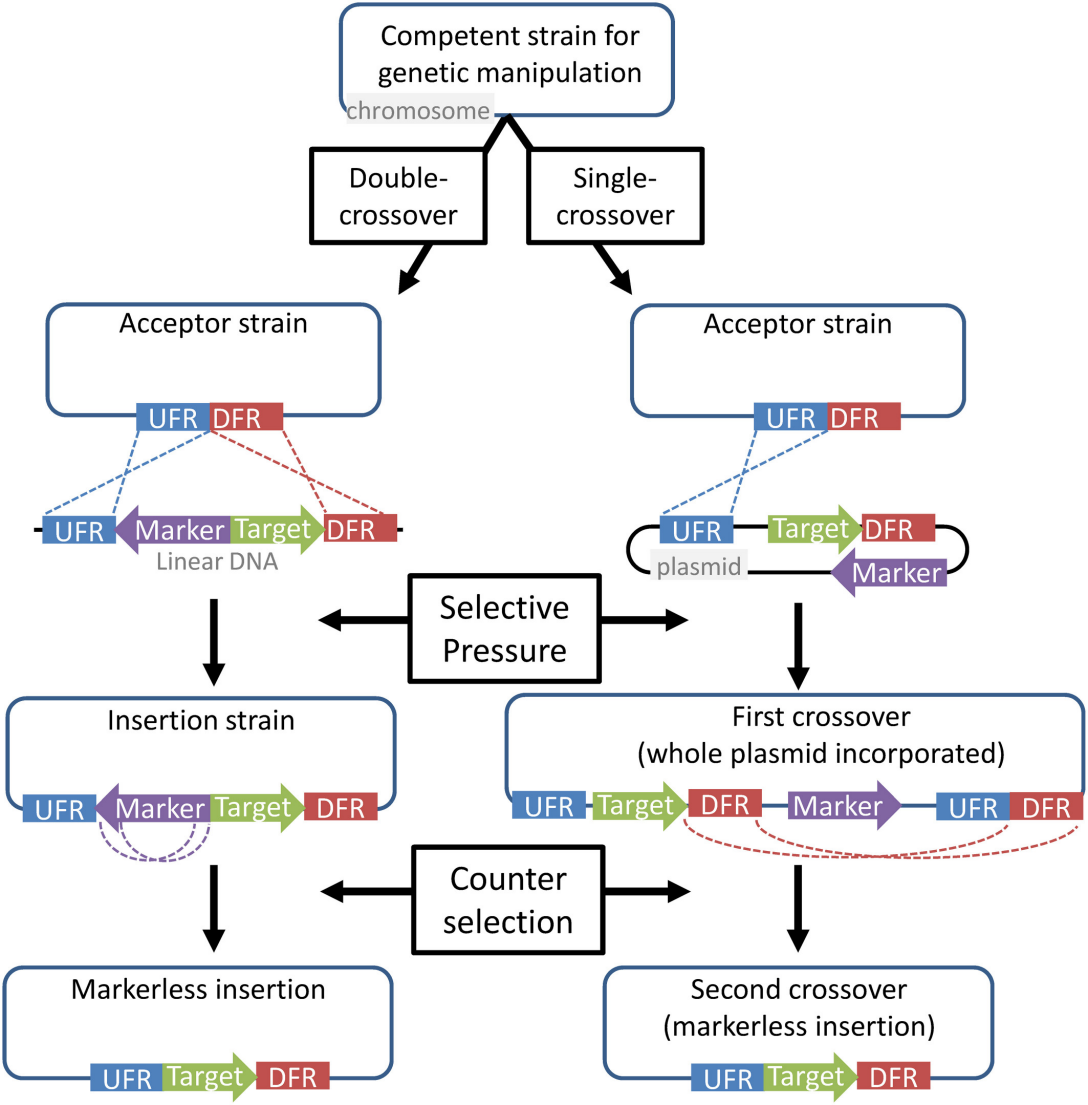
**General strategy for chromosomal gene insertion, used in most of the organisms discussed here**. Upstream flanking regions (blue) and downstream flanking regions (red) are used to direct DNA for the insertion of a target gene (green). A marker gene (purple) provides resistance to a selective pressure, such as the addition of an antibiotic or the absence of an essential nutrient. In the case of a single first-crossover (from a circular plasmid), counter-selection results in a second crossover with the other set of homologous regions, resulting the loss of the plasmid backbone. With double-crossover, short homologous regions flanking the marker allow its removal. In either case, counter-selection recovers a markerless acceptor strain that can be used for subsequent insertions. This method can also be used for gene knockouts.

Most genetic manipulations in extreme thermophiles are chromosome-based rather than plasmid-based. This is due primarily to a lack of reliable replicating vectors in many organisms, and partly to the higher prevalence of nutritional selection, which is less amenable to long-term maintenance of plasmids. Chromosomal modifications are preferable for generating an industrial host, because they provide greater strain stability and eliminate the need for continued selective pressure. Chromosomal insertions and deletions are directed by, and dependent on, homologous flanking regions. These homologous flanking regions guide the marker along with or to the target gene and transform the chromosome. This can occur via either single or double homologous recombination, after which the selective marker can be removed in another recombination event using counter-selection (Figure 2). Several extreme thermophiles, including *P. furiosus, Sulfolobus* species, and *T. kodakarensis*, can be transformed using either single crossover (circular DNA) or double crossover (linear DNA) homologous recombination, while others, such as *C. bescii*, are limited to single crossover events.

## Candidates for high-temperature metabolic engineering

While the list of extremely thermophilic microorganisms available in pure culture has expanded considerably over the past four decades, only a small subset have been characterized in detail from physiological and genomic perspectives. This group has been the focus of efforts to develop molecular genetics tools, driven mostly by a desire to study basic physiological issues. However, the tools that have been developed open up opportunities for metabolic engineering, although only limited results are available to date on this aspect. Table 2 summarizes the key features of extreme thermophiles for which genetics systems have been described. These microorganisms are discussed in detail below.

**Table 2:** **Extreme thermophiles with functional genetic systems, and successful metabolic engineering efforts**.

**Organism (Domain)**	**T_opt_**	**Genetic tools**	**Metabolism**	**Metabolic engineering (titer)**	**Sources**
*Thermoanaerobacter mathranii* (Bacteria)	70°C	Kanamycin Electroporation	Anaerobe Heterotroph (carbohydrates)	Ethanol (2.3 g/L)	Larsen et al., 1997^a^; Yao and Mikkelsen, 2010a^b, c^
*Thermus thermophilus* (Bacteria)	70°C	Kanamycin, uracil/5-FOA Natural competence	Aerobe Heterotroph (carbohydrates or peptides) Denitrification (some strains)	Protein overexpression	Oshima and Imahori, 1974^a^; Tamakoshi et al., 1999^b^; Hashimoto et al., 2001^b^; Moreno et al., 2005^c^; Cava et al., 2009^a^
*Metallosphaera sedula* (Crenarchaea)	75°C	Uracil/5-FOA Electroporation	Aerobe Heterotroph (peptides) Autotroph (S^0^, sulfidic ores, or H_2_)	–	Huber et al., 1989^a^; Maezato et al., 2012^b^
*Caldicellulosiruptor bescii* (Bacteria)	75°C	Uracil/5-FOA Electroporation, restriction enzyme deletion	Anaerobe Heterotroph (cellulose, hexose and pentose sugars)	Ethanol (0.6 g/L) Increased H_2_	Svetlichnyi et al., 1990^a^; Yang et al., 2010^a^; Chung et al., 2013^b^; Cha et al., 2013^c^; Chung et al., 2014^c^
*Sulfolobus acidocaldarius* (Crenarchaea)	80°C	Uracil/5-FOA Sugar inducible promoters Shuttle vector, uracil, β-gal screen	Aerobe Heterotroph (starch, peptides, some monosaccharides)	–	Brock et al., 1972^a^; Grogan, 1989^a, d^, Berkner et al., 2007, 2010; Wagner et al., 2012^b^
*Sulfolobus islandicus* (Crenarchaea)	78°C	Uracil/5-FOA, agmatine, simvastatin Sugar inducible promoters	Aerobe Heterotroph	–	Zillig et al., 1993^a^, Deng et al., 2009; Peng et al., 2012; Zheng et al., 2012; Zhang et al., 2013^b^; Albers and Siebers, 2014^d^
*Sulfolobus solfataricus* (Crenarchaea)	80°C	Lactose, agmatine Sugar inducible promoters	Aerobe Heterotroph (peptides, many mono and polysaccharides)	Cellulose degradation Protein overexpression.	Zillig et al., 1980^a^; Grogan, 1989^a, d^; Worthington et al., 2003^b^; Albers et al., 2006^c^; Berkner et al., 2007^b^; Zhang et al., 2013^b^; Girfoglio et al., 2012^c^
*Thermotoga maritima, T. sp. RQ7, RQ2* (Bacteria)	80°C	Kanamycin Shuttle vector, electroporation, liposomes RQ7 natural competence	Anaerobe Heterotroph (carbohydrates)	Cellulase expression in *RQ2*	Huber et al., 1986^a^, Han et al., 2012, 2014^b^; Xu et al., 2015^c^
*Thermococcus kodakarensis* (Euryarchaea)	85°C	Uracil/5-FOA, tryptophan/6-MP, simvastatin Natural competence β-glycosidase screen	Anaerobe Heterotroph (carbohydrates, peptides with S^0^ reduction)	Protein expression and secretion Increased H_2_	Atomi et al., 2004^a^, Sato et al., 2005; Matsumi et al., 2007; Santangelo et al., 2010^b^, 2011^c^; Takemasa et al., 2011^c^
*Pyrococcus furiosus* (Euryrchaea)	100°C	Shuttle vector, simvastatin, chemical competence (CaCl) and cold shock Uracil/5-FOA Natural competence	Anaerobe Heterotroph (carbohydrates, peptides with S^0^ reduction)	Lactate (0.3 g/L) 3HP (0.3 g/L) Ethanol (2 g/L) Butanol (0.1 g/L)	Fiala and Stetter, 1986^a^; Waege et al., 2010; Lipscomb et al., 2011^b^, Basen et al., 2012, 2014; Hawkins et al., 2015; Keller et al., 2015^c^

a *Isolation/metabolism*.

b *Genetic methods*.

c *Metabolic engineering*.

d *T_opt_*.

### Thermococcus kodakarensis

Originally isolated from a marine solfatara and named *Pyrococcus kodakaraensis*, this euryarchaeon was reported to have an optimum growth temperature of 95°C (Morikawa et al., 1994). However, it was later shown to grow optimally at 85°C and was re-classified as *Thermococcus kodakaraensis* (Atomi et al., 2004; now spelled *T. kodakarensis*). *T. kodakarensis* is an anaerobic heterotroph that grows well on carbohydrates and can utilize peptides if elemental sulfur (S^0^) is present (Atomi et al., 2004). It is well established as a source for archaeal and thermophilic proteins, with nearly 100 genes characterized by the early 2000s (Imanaka and Atomi, 2002). In the past decade, a genetic system has been developed, optimized, and used to investigate a variety of questions about the basic biology of thermophiles and archaea. Indeed, while the development of genetic tools for organisms that can grow above 80°C was pioneered in *T. kodakarensis* by Atomi, Imanaka and coworkers (as outlined below), so far little work has been done to create metabolically engineered *T. kodakarensis* strains of industrial interest.

#### *T. kodakarensis* genetics

The first demonstration of targeted gene disruption used a uracil auxotrophic strain created by exposure to 5-FOA, allowing the *pyrF* gene to be used as a selectable marker for disruption of another gene (*trpE*) by homologous recombination (Sato et al., 2003). Soon after this initial report, a complete genome sequence of *T. kodakarensis* was published (Fukui et al., 2005). Over the next decade extensive work was carried out to optimize genetic techniques for *T. kodakarensis* (Santangelo and Reeve, 2011; Hileman and Santangelo, 2012). In summary, *T. kodakarensis* is naturally competent for transformation by homologous recombination with either linear or circular DNA, which can be introduced via *E. coli* shuttle vector. Selection can be accomplished with nutrient auxotrophy for uracil, tryptophan, arginine (citrulline), and agmatine, and antibiotic sensitivity to mevinolin/simvastatin, while counter-selection is possible with 5-FOA and the adenine analog 6-methylpurine (6-MP). More recent studies involving genetic manipulation of *T. kodakarensis* have continued to rely on methods laid out in the reviews referenced above, which have also been successfully applied in related species such as *Thermococcus onnurineus* (Kim et al., 2013). The focus has now shifted away from development of the genetic system to applying these mature techniques to answer a variety of scientific questions.

Examples of the extent to which genetic modification is possible in *T. kodakarensis* include one investigation that generated 13 different deletion strains, including double-deletion mutants which required the use of 6-MP counter-selection (Santangelo et al., 2011), and another that involved 14 different strains and included one with four rounds of *pyrF*/5-FOA selection/counter-selection (Harnvoravongchai et al., 2014). Additional metabolic engineering tools available in *T. kodakarensis* include the strong constitutive promoters of glutamate dehydrogenase (Matsumi et al., 2007) and cell surface glycoprotein (Mueller et al., 2009) for protein overexpression, and a signal peptide that enables protein secretion (Takemasa et al., 2011).

#### *T. kodakarensis* metabolic engineering

*T. kodakarensis* has been metabolically engineered for improved hydrogen production, resulting in a strain capable of producing several times more hydrogen than wild-type (Santangelo et al., 2011). Efforts to further improve hydrogen production are ongoing (Kanai et al., 2015), and *T. kodakarensis* shows promise as a bio-hydrogen production platform. Strains optimized for overexpression and secretion of enzymes have also been developed (Takemasa et al., 2011). There are currently no reports of expressing full heterologous metabolic pathways in *T. kodakarensis*, but it has been used to produce recombinant versions of proteins from other thermophiles for study when expression in *E. coli* yielded inactive protein (Mueller et al., 2009). *T. kodakarensis* expresses a natural viral defense system in the form of several CRISPR cassettes (Grissa et al., 2007). Strains have been engineered to use the CRISPR system to target specific sequences of foreign DNA (Elmore et al., 2013), opening up the potential to protect industrial strains from problematic viruses.

### Pyrococcus furiosus

Robust and fast-growing, *Pyrococcus furiosus* is convenient to work with, and also happens to be the highest temperature organism for which a versatile genetic system is available. Able to grow on a variety of peptide and carbohydrate substrates, *P. furiosus* is a natural hydrogen producer, and is also capable of reducing elemental sulfur (S^0^). It has already been genetically engineered to express a variety of heterologous metabolic pathways, despite the fact that the first reports of a functional genetic system did not appear until 2010.

*P. furiosus* was isolated from a shallow marine solfatara and found to have an optimum temperature of 100°C (Fiala and Stetter, 1986). It is a euryarchaeal heterotroph capable of growth on peptides and some oligo- and polysaccharides. Sulfur (S^0^) is required for peptide utilization, resulting in the production of organic acids and H_2_S as byproducts. Carbohydrates can be utilized either with S^0^ or without, where reducing equivalents are disposed of as H_2_ (Adams et al., 2001). *P. furiosus* and *T. kodakarensis* appear to share a virtually identical system of reductant disposal, where the highly homologous multi-subunit membrane complex proteins Mbh and Mbx are responsible for H_2_ and H_2_S production, respectively (Schut et al., 2013).

#### *P. furiosus* genetics

The genetic system in *P. furiosus* has benefited from successes and developments made previously in *T. kodakarensis*. In fact, the first successful genetic manipulation in *P. furiosus*, which used simvastatin selection to allow for protein expression from a shuttle vector (Waege et al., 2010), borrowed a transformation protocol (using divalent cations and heat-shock) developed for *T. kodakarensis* (Sato et al., 2003). A subsequent effort applied counter-selection via 6-MP to perform a single nucleotide deletion (Kreuzer et al., 2013). These are the only reports of genetic transformations in wild-type *P. furiosus*, the majority of genetic manipulations have been based on the discovery of a naturally competent strain COM1 (Lipscomb et al., 2011).

Compared to the wild-type *P. furiosus* strain, COM1 has undergone several large-scale chromosomal rearrangements. However, less than 2% of genes (36 ORFs) experienced changes that reduced sequence identity with their WT homologs below 90%, and no differences in metabolic phenotype were found (Bridger et al., 2012). The original report of COM1 transformation used uracil/5-FOA selection to generate a double-deletion mutant. The uracil auxotroph for this transformation was generated by simvastatin selection, while agmatine (Hopkins et al., 2011) and tryptophan (Farkas et al., 2012) prototrophic selection have also been demonstrated. Most recent work has continued to rely on the simpler uracil/5-FOA system. Promoters available for protein overexpression include the strong constitutive promoter of the S-layer protein (*P*_*slp*_) (Hopkins et al., 2011), and the cold-inducible promoter from cold-shock protein A (*P*_*cipA*_) (Basen et al., 2012). Recently, entire operons up to 17 kilobases long have been transformed into *P. furiosus* using bacterial artificial chromosomes (Basen et al., 2014; Lipscomb et al., 2014).

#### *P. furiosus* metabolic engineering

As with most new genetic systems, the first demonstrations of genetic modification in *P. furiosus* involved gene knockouts. The genes encoding two soluble hydrogenases, believed to be essential to *P. furiosus* metabolism, were deleted in the original report of COM1, although there was no change in phenotype under the usual laboratory conditions (Lipscomb et al., 2011). The ancestral ability of *P. furiosus* to utilize chitin was recovered by a single base deletion (Kreuzer et al., 2013). But the most impressive examples of metabolic engineering in this organism have been in the expression of heterologous pathways.

The core metabolic strategies available at the extreme temperatures where *P. furiosus* grows best appear to be rather limited. For example, alcohol production is very rare among extreme thermophiles, possibly because the archaea, which predominate at high temperatures, are not known to produce significant amounts of any alcohol naturally (Basen et al., 2014). Therefore, heterologous pathways to be inserted into *P. furiosus* must be taken from less thermophilic organisms, requiring that they be expressed under what amounts to cold-shock conditions for *P. furiosus*, which maintains some degree of growth down to temperatures as low as 70°C (Weinberg et al., 2005). For example, addition of a lactate dehydrogenase from *Caldicellulosiruptor bescii* (T_opt_ 75°C), under the control of *P*_*cipA*_, allowed production of the non-native product lactate to a titer of 0.3 g/L at 72°C (Basen et al., 2012). Another single-enzyme insertion gave *P. furiosus* the ability to produce ethanol via a novel pathway also incorporating native enzymes, reaching titers of 2 g/L (Basen et al., 2014).

More complex multi-enzyme metabolic engineering has also been demonstrated in *P. furiosus*. A three-enzyme pathway (involving five genes) constituting part of the carbon fixation cycle of *Metallosphaera sedula* (T_opt_ ~75°C) was expressed heterologously in *P. furiosus*, yielding titers of the industrially relevant chemical 3-hydroxypropionate (3HP) of 0.05 g/L at 72°C (Keller et al., 2013). Deletion of a competing pathway roughly doubled 3HP titers over the parent strain (Thorgersen et al., 2014), while additional accessory enzymes and improved bioreactor conditions increased 3HP titers to 0.3 g/L (Hawkins et al., 2015). A synthetic butanol pathway consisting of six genes from three thermophilic bacteria enabled *P. furiosus* to produce butanol at 60°C, but titers remained low (0.07 g/L) even in 200x concentrated cell-suspensions (Keller et al., 2015). Heterologous expression of the massive 17 kb, 18-gene formate dehydrogenase operon from *Thermococcus onnurineus* allowed *P. furiosus* to generate H_2_ from formate (Lipscomb et al., 2014). Insertion of another large operon from *T. onnurineus*, encoding the carbon monoxide dehydrogenase complex, conferred the ability of *P. furiosus* to use CO as a source of reductant (Basen et al., 2014).

With an incredible diversity of functional engineered pathways, *P. furiosus* is the greatest success story so far in metabolic engineering of extreme thermophiles. However, additional improvements will be necessary to turn current strains, which often produce only trace amounts of the target chemicals, into viable industrial hosts. Some progress has already been made in this area, particularly in 3HP production, where additional enzymes and improved growth conditions increased titers nearly ten-fold (Hawkins et al., 2015).

### Sulfolobus species

Members of the archaeal genus *Sulfolobus* are found in a variety of acidic freshwater hot springs with water temperature around 80°C and pH below 3, making them extreme thermoacidiphiles. Three *Sulfolobus* species, *S. acidocaldarius, S. solfataricus*, and *S. islandicus*, have functional genetic systems. All three species are obligate aerobes, grow well on rich media, and single colonies can be isolated on solid substrates. Combined with their genetic tractability, these traits make them excellent model organisms. *Sulfolobus* species have been used extensively to elucidate the mechanisms and cellular machinery of transcription in archaea, as a model host for archaeal viruses, and as a source of easily crystallized thermophilic proteins. So far, no member of *Sulfolobus* has been metabolically engineered to produce a commercially-desirable chemical product.

Following the initial isolation of *S. acidocaldarius* (Brock et al., 1972) and *S. solfataricus* (de Rosa et al., 1975), species were regularly re-isolated independently (Zillig et al., 1980). This, combined with difficulties obtaining pure cultures, led to the use of mixed and misidentified strains during the early stages of *Sulfolobus* research (Grogan, 1989). *S. islandicus* was isolated more recently (Zillig et al., 1993), but has suffered from similar confusion: fresh isolates are reported so frequently that no single strain has been dominant enough to be thoroughly characterized; in fact, the species name *islandicus* is not yet officially recognized (Zuo et al., 2014).

The first isolation report for a *Sulfolobus* species described its ability to grow autotrophically by oxidation of sulfur (Brock et al., 1972); subsequently, autotrophic *Sulfolobus* sp. have been re-isolated from the environment (Wood et al., 1987; Nixon and Norris, 1992). However, current laboratory strains of *S. acidocaldarius* and *S. solfataricus* appear to have lost this ability, and only grow if organic substrates are present (Berkner and Lipps, 2008). A comparison of the two strains during heterotrophic growth indicates that, while both grow well on complex protein sources and starch, *S. solfataricus* can utilize a much more diverse set of mono- and disaccharides than *S. acidocaldarius* (Grogan, 1989). The original *S. islandicus* isolates were determined to be obligate heterotrophs, growing well on complex media containing tryptone or yeast extract (Zillig et al., 1993), but no detailed characterization of preferred energy sources has been done.

#### *Sulfolobus* genetics

The first genetic modifications in *Sulfolobus* relied on nutrient selection. Lactose selection (Worthington et al., 2003) is limited to *S. solfataricus* and *S. islandicus*, because *S. acidocaldarius* cannot be cultured on lactose-based minimal media. Uracil selection, which has been used in all three species (Albers et al., 2006; She et al., 2009; Wagner et al., 2012), allows for richer media and better cell growth than lactose, but suffers from high background due to residual uracil, especially in *S. solfataricus* and *S. islandicus* (Berkner and Lipps, 2008). Genetic transformations have also been accomplished in *S. islandicus* with simvastatin selection (Zhang and Whitaker, 2012). Electroporation is the standard means of transformation across all three species. DNA methylation is used in some methods involving *S. solfataricus* (Albers and Driessen, 2008) and *S. acidocaldarius* (Wagner et al., 2012), but does not seem to be necessary for *S. islandicus*.

Genome sequences are available for the type strain of *S. acidocaldarius* (DSM 639) (Chen et al., 2005) and *S. solfataricus* strain P2 (DSM 1616) (She et al., 2001), while nearly 20 different strains of *S. islandicus* have been sequenced. None of the *islandicus* strains are available from culture collections and no type strain has been designated (Zuo et al., 2014). *Sulfolobus* species, particularly *S. islandicus*, have been isolated in conjunction with viruses (Guo et al., 2011), and have played an important role in understanding the diversity and host interactions of archaeal viruses (Zillig et al., 1993; Greve et al., 2004; Bize et al., 2009; Prangishvili, 2013). As a result, a variety of viral-based vectors are available for genetic manipulation (Aucelli et al., 2006; Berkner et al., 2007; Berkner and Lipps, 2008). Inducible promoter systems are available (Berkner et al., 2010; Peng et al., 2012), and a beta-galactosidase based reporter system (Jonuscheit et al., 2003) has been utilized in all three species.

#### *Sulfolobus* metabolic engineering

While production of novel products has yet to be demonstrated in *Sulfolobus*, an *S. sulfataricus* strain that utilizes cellulose more effectively has been created by overproduction of a native endoglucanase (Girfoglio et al., 2012). Therefore, if product pathways are developed, *S. sulfataricus* could be engineered to utilize desirable complex biomass sources. There are many other examples of homologous or heterologous overexpression of proteins in *Sulfolobus*, sometimes because the protein cannot be expressed in functional form otherwise, but often simply as demonstration of a new method of genetic modification. Genetic system development in *Sulfolobus* has been underway for over a decade, but remains a research focus.

### Thermus thermophilus

First isolated from a Japanese hot spring in 1974, *Thermus thermophilus* is an aerobic bacterium that grows best between 70°C (Oshima and Imahori, 1974; Williams et al., 1995) and 80°C (Swarup et al., 2014). Like other thermophiles, *Ts. thermophilus* has served as a source of crystallizable proteins, with structures available for over 20% of the proteins represented in its genome (Swarup et al., 2014). *Ts. thermophilus* is especially capable for natural DNA uptake (Koyama et al., 1986; Hidaka et al., 1994), and as such has been used to study the cellular machinery involved in natural competence (Schwarzenlander and Averhoff, 2006; Salzer et al., 2015a,b).

*Ts. thermophilus* grows aerobically on sugars and peptides (Oshima and Imahori, 1974), and is also capable of utilizing lipids and triglycerides (Leis et al., 2014). Additionally, strain HB8 can grow anaerobically by denitrification (Cava et al., 2009). *Ts. thermophilus* appears to have a metabolic efficiency comparable to *E. coli*, achieving similar biomass yields during growth on minimal glucose medium, albeit with a longer doubling time of approximately 3 h (Swarup et al., 2014).

#### *Ts. thermophilus* genetics

Genome sequences are available for *Ts. thermophilus* strains HB8 and HB27, consisting of a slightly less than 2 Mb chromosome, a 200 kb megaplasmid, and a second 9 kb plasmid in HB8 (Henne et al., 2004; Brüggemann and Chen, 2006). The HB8 strain appears to be polyploid, like the closely related *Deinococcus radiodurans*, with cells able to maintain two different antibiotic resistance genes at the same location on the chromosome (Ohtani et al., 2010). The presence of multiple chromosome copies was proposed to present a significant challenge to genetic manipulation, but more recent reports of genetic modification in strain HB27 (Leis et al., 2014; Carr et al., 2015) make no mention of it.

Early genetics work in *Ts. thermophilus* relied on thermostable kanamycin resistance genes for plasmid cloning and gene knockouts, but thermostable genes for resistance to the antibiotics hygromycin and bleomycin have since been developed (Cava et al., 2009). Markerless deletions can be accomplished by the uracil/5-FOA method (Tamakoshi et al., 1999), or by a recently reported method entailing use of a phenylalanine analog and a mutated tRNA gene that confers sensitivity to it (Carr et al., 2015).

#### *Ts. thermophilus* metabolic engineering

The genetic system of *Ts. thermophilus* is facile enough to generate quadruple-knockout strains (Leis et al., 2014), and has been used to overexpress active tagged versions of its own proteins (Hidalgo et al., 2004; Moreno et al., 2005). So far, the only metabolic engineering reported in *Ts. thermophilus* involved transferring the ability to grow anaerobically by denitrification to previously obligately aerobic strains (Ramírez-Arcos et al., 1998).

## Early stage genetic systems for extreme thermophiles

### Metallosphaera sedula

*M. sedula*, a relative of the *Sulfolobus* species discussed above, is an extreme thermoacidophile, growing optimally at 75°C and pH of around 3 (Huber et al., 1989). It is an aerobe, capable of autotrophic growth by oxidizing sulfidic ores or hydrogen, heterotrophic growth on peptides, or a combination of the two (Auernik and Kelly, 2010). The genome sequence has been published (Auernik et al., 2008). So far there is only one report on development of a genetic system, which used uracil/5-FOA and electroporation to knockout a gene involved in *M. sedula*'s surprisingly high tolerance to metal ions (Maezato et al., 2012). Despite current limitations to the genetic system, *M. sedula* shows promise as an industrially relevant strain because of its highly versatile native metabolism. Its uniquely archaeal carbon fixation pathway (Kockelkorn and Fuchs, 2009), coupled with the ability to grow on hydrogen gas, makes *M. sedula* a promising candidate for eventual production of electrofuels (Hawkins et al., 2013), while the ability to solubilize highly refractory chalcopyrite ores makes it a candidate for use in high-temperature bioleaching operations (Zhu et al., 2013).

### Thermoanaerobacter mathranii

*T. mathranii* is an extremely thermophilic anaerobic bacterium that was isolated from an Icelandic hot spring. It grows optimally at 70–75°C on xylose and produces primarily ethanol (~20 mM) and acetate (~13 mM) (Larsen et al., 1997). *T. mathranii* garnered biotechnological interest when it was found to grow on, and produce ethanol from, lignocellulosic biomass at high temperatures (Ahring et al., 1999). While this ability to use lignocellulosic biomass is appealing, improving its ethanol yield is necessary to exploit *T. mathranii* for biofuel production. To accomplish this, glycerol dehydrogenase from *Thermotoga maritima* was expressed in *T. mathranii*, while knocking out lactate dehydrogenase using kanamycin resistance as a selectable marker. The result is ablation of lactate production and an increase of 19% in ethanol yield (Yao and Mikkelsen, 2010b).

### Caldicellulosiruptor bescii

Recently re-classified from its original designation as *Anaerocellum thermophilum, C. bescii* grows optimally at 75°C, and is capable of utilizing a variety of cellulosic substrates (Svetlichnyi et al., 1990; Yang et al., 2010). It has a published genome sequence (Kataeva et al., 2009). The development of a genetic system in this organism is in the beginning stages, based on uracil/5-FOA and electroporation (Chung et al., 2013). One added challenge has been the presence of restriction enzymes, necessitating either the use of methylated transformation constructs, or strains that lack the relevant enzymes (Chung et al., 2012, 2013). Despite its recent development, the genetic system has already been used to metabolically engineer *C. bescii* strains that produce ethanol (0.6 g/L) (Chung et al., 2014), and to increase hydrogen production by deleting an alternative reductant disposal pathway (Cha et al., 2013). In addition, a heterologous gene encoding an archaeal tungsten-containing enzyme was successfully expressed in *C. bescii*, thereby demonstrating that the organism could assimilate tungsten, a metal rarely used in biological systems (Scott et al., 2015).

### Thermotoga

Members of the genus *Thermotoga*, including *T. maritima* (Huber et al., 1986) and *T. neapolitana* (Jannasch et al., 1988), have been isolated from various marine geothermal vents. With optimum growth temperatures around 80°C, *Thermotoga* species are among the most thermophilic bacteria known. Members of the genus contain a remarkable number of sugar utilization genes, allowing for growth on a wide variety of carbohydrates (Chhabra et al., 2003; Conners et al., 2005; Frock et al., 2012). The early publication of the genome sequence of *T. maritima* (Nelson et al., 1999) cemented its status as a model hyperthermophile. A plasmid isolated from *Thermotoga* strain *RQ7* was used to transform *T. maritima* and *T. neapolitana* with antibiotic resistance to chloramphenicol or kanamycin, but the plasmids were gradually lost even with continued selective pressure (Yu et al., 2001). Ten years later, *T. maritima* and *T*. sp. *RQ7* were finally stably transformed with an *E. coli* shuttle vector conferring kanamycin resistance (Han et al., 2012). Transformation has been accomplished with liposomes (Yu et al., 2001) and electroporation (Han et al., 2012), but some strains are also naturally competent (Han et al., 2014). The only demonstration of metabolic engineering thus far involved recombinant expression of cellulases from *Caldicellulosiruptor saccharolyticus*, which were fused with native signal peptides for export, leading to cellulolytic activity in the cell supernatant; however, expression was not stable long term (Xu et al., 2015).

## Overview of current state of industrial bioprocessing

In order to understand the potential of extreme thermophiles for bioprocessing, as well as how far they still have to go, it is worth considering the current state of the art, which is still reliant on mesophilic hosts. While many chemicals and fuels of interest have been successfully created in microorganisms, production has been hindered by a few key factors. One of the most notable can be attributed to the high proportion of water necessary for all biological processes. Thus, separating low concentrations of the target molecule from massive quantities of water requires significant energy inputs for heating, cooling, distillation, and transportation. The low concentration of reactant (and product) inherent in biological processes also leads to higher capital costs, since reactors, tanks, piping, and pumps for a process that is 10% reactant and 90% water must be ten-times larger than for a comparable petrochemical process stream that approaches 100% reactant. Another hurdle to renewable chemical production is the high cost of feedstocks, although, as shown in Table 3, U.S. corn and Brazilian sugar are now available at rates competitive with crude oil. Both corn and crude oil provide energy at less than $10/GJ. Various other feedstocks with potential for use by biological organisms, such as hydrogen and natural gas, are also available at competitive rates.

**Table 3:** **Commodity prices of fuel feedstocks, including common biomass, and fossil-derived sources**.

**Energy Source**	**Cost (Commodity Market Units)**	**Cost ($/mt)**	**Energy Density (MJ/kg)**	**ΔH_*c*_ ($/GJ)**
Powder River Basin Coal (Wyoming)	$11.55/ton^a^	$13	10.2^a^	$1.25
Natural Gas	$2.71/MMBTU^b^	$144	56.1^b^	$2.57
Central Appalachian Coal	$49.95/ton^a^	$55	14.5^a^	$3.79
Crude Oil (WTI)	$43.22/bbl^c^	$321	42.7^j^	$7.52
Corn Stover	$75/mt^d^	$75	8.7^j^	$8.61
Corn Starch	$3.72/bu^e^	$146	16.5^k^	$8.85
Hydrogen Gas	$1470/mt^f^	$1470	120^k^	$12.25
Brazilian Sugar	$262/mt TRS^g^	$262	16.5^k^	$15.90
Electricity	$0.0747/kWh^h^	–	–	$20.75
Carbon Monoxide	$240/mt^i^	$240	10.1^l^	$23.76

*Energy content as lower heating value, except electricity*.

a *Spot prices from EIA website, August 2015*.

b *Henry Hub spot prices from EIA website, August 2015*.

c *Cushing, OK spot prices from EIA website, August 2015*.

d *Prices received for stover delivered to POET-DSM plant, Emmetsburg, IA, Sept 2014*.

e *Yellow dent corn spot price per Chicago Board of Trade (Assumptions: Corn starch 75 wt% of bushel and 20% discount for DDG credit)*.

f *Hydrogen production from natural gas (Clean Energy States Alliance)*.

g *World Bank Sugar Monthly Price (Index Mundi, July 2015, no by-product credit)*.

h *EIA June 2015 reported industrial electricity costs, West North Central average (IA, KS, MN, MO, NE, ND, SD)*.

i *Estimate (production cost) from moderate scale on-site Calcor Process (2001 Report by DNV)*.

j *Oak Ridge National Lab list of heating values for gases, liquids, and solids*.

k *Values of heats of combustion*.

l *Heating Value of Gases, EIA*.

In the landscape of chemicals being targeted for production via biological organisms, ethanol stands alone as the only chemical to have rivaled an industrial commodity on a volume and economic basis. Over 14 billion US gallons (53 billion liters) of ethanol were produced in the United States in 2014, mostly from corn starch, with Brazil adding over six billion gallons (23 billion liters) from sugar (RFA, 2014). No other biologically produced chemical or fuel has approached the one billion gallon mark. Ethanol benefits from a number of advantages, especially the natural ability of yeast to metabolize sugars to ethanol at high titers and with high efficiency, thus avoiding the need for extensive genetic engineering. However, the success of ethanol proves that bio-based chemical production at scale is possible, and recent progress by industry startups generating a variety of other chemicals (Table 4) confirms this.

**Table 4:** **Commercial scale biochemical production (excluding 1st generation ethanol)**.

**Company**	**Chemical**	**Organism**	**Nameplate capacity (1000 metric tons)**	**Startup (yr)**	**Facility location**
Beta Renewables	Ethanol (cellulosic)	NR	40	2013	Crescentino, Italy
DSM-Poet	Ethanol (cellulosic)	Yeast	60	2014	Emmetsburg, IA
Abengoa	Ethanol (cellulosic)	Yeast	75	2014	Hugoton, KS
Dupont	Ethanol (cellulosic)	Zymomonas mobilus	82	2015	Nevada, IA
Lanzatech	Ethanol (from waste gas)	Clostridium autoethanogenum	47	2017	Ghent, Belgium
Cargill^a^	Lactic acid	NR	180	2002	Blair, NE
Dupont Tate & Lyle	1,3-propanediol	*Escherichia coli*	63	2006	Loudon, TN
Genomatica	1,4-butanediol	*Escherichia coli*	30	2015	Adria, Italy
Gevo	Isobutanol	Yeast	55	2012	Luverne, MN
Myriant	Succinic acid	*Escherichia coli*	14	2013	Lake Providence, LA
BioAmber	Succinic acid	Yeast	30	2015	Sarnia, Ontario, Canada
Elevance	C10-C18 oils, acid, olefins	Abiotic catalyst	180	2013	Gresik, Indonesia
Amyris	Farnesene, farnesane	Yeast	33	2012	Brotas, Brazil
Solazyme	Custom oils	Microalgae	20	2014	Clinton, IA

*Actual plant startup production typically falls well below nameplate capacity. NR, not reported*.

a *Vink et al. (2004) Other sources: company websites, press releases, and patents*.

A joint venture between Dupont and Tate & Lyle was the first to achieve a scale in the thousands of metric tons per year of a commodity chemical using a metabolically engineered host. Production of 1,3-propanediol from corn starch commenced in 2006 and, nearly a decade later, the company reports progress on expanding production. Recent years have brought more commercial facilities onto the scene (Table 4), representing billions of dollars in capital investment. The move from demonstration to commercial-scale shows that the investment community is optimistic about the future prospects of biological chemical production. While none of the examples in Table 4 uses a thermophilic host, most of the research on these commercial scale projects was started a decade ago, before significant tools were available for genetic manipulation of thermophiles. The experiences gained in these initial projects may provide further evidence of the advantages of thermophilic processes.

## Future of extremely thermophilic metabolic engineering: Challenges and promise

The genetic systems for several species of extreme thermophiles are now advanced enough to begin developing metabolically engineered strains to produce industrially relevant chemicals, but so far only *P. furiosus* has seen significant progress in this area. Thermophiles research has historically been focused on answering the basic questions of how life functions at high temperature: how do transcription and translation occur, how do protein molecules fold, how can metabolism proceed through thermolabile intermediates, and could the earliest life have evolved at high temperatures? However, the emerging sector of industrial biotechnology has added the additional focus of producing fuels and chemicals by exploiting thermophilicity. Some thoughts on this subject follow.

### Process control

**Figure 3:**
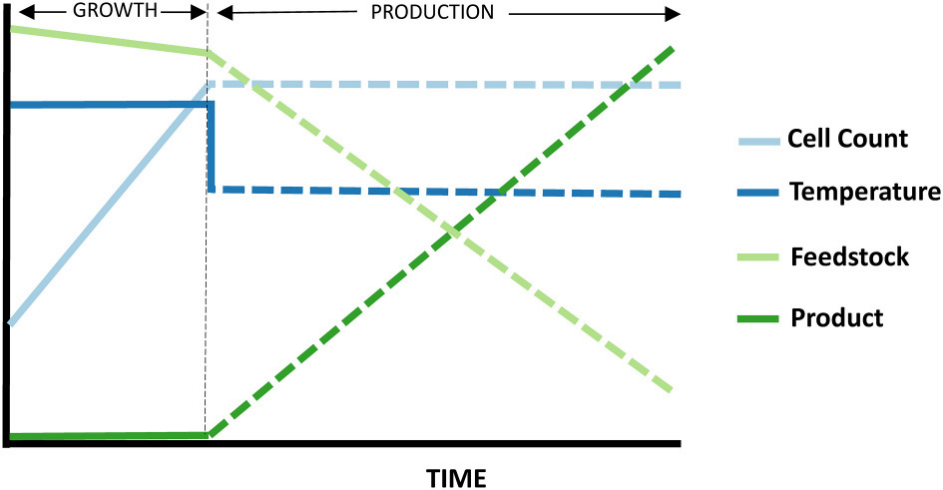
**Temperature-shift strategy involving a hyperthermophilic host expressing more moderately thermostable recombinant enzymes**. Reduced temperatures result in a transition from growth to production phase. The hosts enzymes, naturally optimized for higher temperatures, become less active, and the cell growth rate stalls. Meanwhile, the recombinant enzymes from less thermophilic sources then re-fold and begin producing chemicals. Enzyme production can furthermore be coupled to temperature shift through the use of cold-induced promoters.

A number of advantages become available when working with a thermophilic host. One that has already been applied in *P. furiosus* (Basen et al., 2012; Keller et al., 2013) is the use of temperature to regulate expression of recombinant enzymes, allowing for a shift from growth phase (where substrate goes to increase cell biomass) to production phase, where substrate is primarily being converted to product, and host metabolism is minimized (Figure 3).

A similar temperature-shift has been performed with *E. coli*, where cells are cooled to 10–15°C prior to expression to improve recombinant protein solubility (Qing et al., 2004). The method is common enough at lab-scale that kits are commercially available, including ArcticExpress (Agilent Technologies, Santa Clara, USA) and pCold (TaKaRa, Otsu, Japan), but the costs of refrigeration make it infeasible for an industrial process. In contrast, a cold-shock expression in thermophiles could be accomplished using ambient water or air as a heat acceptor.

### Contamination

By far the most significant benefit of thermophilic production is expected to be minimized contamination risk. Biorefineries experience two main types of contamination. Chronic, low level infections can reduce yields, but at large scale they are so difficult to prevent that they have been accepted as a fact of life at many corn ethanol plants. Severe infections, which crop up unpredictably, can compromise the viability of the production host, leading to a “stuck fermentation” and requiring a complete shutdown (Skinner and Leathers, 2004).

The harsh pre-treatments sometimes used to solubilize biomass would be expected to eliminate wild-type bacteria that might compete with the production host, but contamination remains a major industrial problem. The load of bacterial contaminants was found to be comparable between two dry-grind ethanol plants that incorporated a high-temperature saccharification pre-treatment, and a wet-mill plant which did not (Skinner and Leathers, 2004). Bacterial load was also not significantly affected by differing degrees of antibiotic use among the three processing plants, although the diversity of bacterial contaminants declined with increased antibiotics. Therefore, having a sterile feedstock or antibiotics in the bioreactor is not enough to prevent opportunistic bacteria from taking advantage of the abundant nutrients and mild conditions found in a bioreactor operated under mesophilic conditions. In contrast, using a thermophilic host ensures that all parts of the plant can be kept at elevated temperatures, so there are no ‘safe’ corners for a persisting reservoir of contaminating microorganisms. The benefits of a contamination-free thermophilic process are difficult to quantitate; production losses due to chronic contamination in ethanol plants have been estimated at anywhere between 2 and 22% (Beckner et al., 2011). The cost of severe contamination events (leading to “stuck” fermentations) is even more difficult to estimate, since there is little information about their frequency in the literature.

The use of an extremely thermophilic host is expected to reduce the risk of bacterial contamination in biorefineries, but the possibility of viral infections remains. Viruses affecting both bacterial and archaeal hyperthermophiles have been identified, although interestingly the archaeal species infected are limited to the crenarchaeoata (Pina et al., 2011; Pietilä et al., 2014). No virus has yet been reported for a member of the hyperthermophilic euryarchaeota, although the presence of a CRISPR system in both *T. kodakarensis* and *P. furiosus* (Grissa et al., 2007) suggests that they do experience viral infections. The native CRISPR system of *T. kodakarensis* was engineered to target specific sequences of foreign DNA, successfully preventing transformation with plasmids containing the target sequences (Elmore et al., 2013). This indicates that *T. kodakarensis*'s CRISPR system could be used to “immunize” industrial strains against problematic viruses in a manner similar to that already implemented in commercially available cheese and yogurt cultures (Dupont/Danisco, 2012; Grens, 2015).

### Energy requirements

One of the oft-cited concerns for the use of thermophiles in industrial fermentations is the energy required to heat the process. There are two major thermal energy requirements for an industrial fermentation: sterilization and fermentor temperature maintenance. As discussed above, sterilization is required for mesophilic fermentations to avoid contamination, and often achieved by heating the fermentation medium to 121°C for a short period (up to 60 min), then cooled back down to the target fermentation temperature. For a thermophilic fermentation, the sterilization process heat inputs would be no higher, and could potentially be reduced; hardy bacterial spores may be able to survive exposure to high temperatures, but they cannot grow at them.

Fermentor temperature maintenance actually provides an opportunity for energy savings if a thermophile is used. All organisms produce heat as a byproduct of metabolic processes. At large scales, the metabolic heat produced outweighs heat lost to the environment through the fermentor walls or evaporation (Yang et al., 2008). As a result, cooling is required to maintain the fermentor at a constant temperature. The cooling duty for a large bioreactor with a high-density culture is extremely high, and can be one of the limiting factors for fermentation scale-up (Yang, 2010). A further difficulty for mesophilic fermentations is the small thermal driving force between the fermentation temperature and the ambient environment, which limits heat removal, often making refrigeration necessary, a further energy cost (Abdel-Banat et al., 2010). The metabolic heat generated, and thus the heat removal required, are primarily dependent on the metabolic activity of the culture and not on the fermentor temperature or organism used (Blanch and Clark, 1997). Thus, a thermophilic fermentation would require a similar amount of heat removal as a mesophilic fermentation. Furthermore, this heat would be much easier to remove because of the large temperature differential between a thermophilic fermenation and the environment, providing the possibility for substantial cost savings (Abdel-Banat et al., 2010).

An additional opportunity for energy savings in thermophilic industrial fermentations is product separation, which can be the most energy intensive part of a process, since it is often carried out at elevated temperatures. In particular, thermophilic production of volatile products, such as fuel alcohols, allows for the possibility of facilitated product removal. The use of thermophilic organisms would be a favorable match for most separations processes recovering volatile products from fermentation broth, including distillation, gas stripping, and pervaporation (Vane, 2008).

### Metabolic engineering potential

While metabolic engineering opens up the possibility of engineering desired chemical pathways into any genetically tractable host, it is worth remembering that *S. cerevisiae*, the current workhorse of bio-ethanol production, came to dominate the field because it was already an excellent ethanol producer. Just because the appropriate enzymes can be inserted into an organism does not mean the resulting mutant will be industrially useful. Therefore, desirable hosts should be selected not only for what they can be engineered to do, but also for what they already do well. Fortunately, the group of extreme thermophiles discussed above exhibit a variety of desirable properties natively. Many are capable of metabolizing a diverse set of sugar polymers and monomers, and *C. bescii* can even degrade unpretreated lignocellulosic biomass (Yang et al., 2009). Coupled with production of ethanol as either a minor or major natural metabolite, this makes them promising candidates for bio-ethanol production from non-food feedstocks. *M. sedula* and the *Sulfolobus* species grow well at low pH, a significant advantage for production of acidic products such as lactic and 3-hydroxypropionic acids, which are easier to purify in their protonated forms (Maris et al., 2004). *M. sedula*'s ability to solubilize metals by oxidizing them has applications in bioleaching, while its novel carbon-fixation pathway (Berg et al., 2007) offers a potential alternative to the RuBisCo-dependent Calvin Cycle for carbon-capture applications.

Fuels are typically highly reduced organic molecules, so various efforts to maximize biofuel titers have focused on tuning the redox pathways within mesophilic hosts to favor the production of reduced end products (Liu et al., 2015). More oxidized products, such as lactic acid for production of biodegradable plastics, can be selected for by shifting metabolism in the other direction (Lee et al., 2015). Substantial progress toward redox-tuning in thermophiles has already been made. The redox pathways of several extreme thermophiles are well understood (Schut et al., 2013), and extensive manipulation of these pathways has been demonstrated in *T. kodakarensis* (Santangelo et al., 2011). The ability to select for the production of alcohols, rather than organic acids, has been demonstrated in recombinant *P. furiosus* by modulating the external redox environment (Basen et al., 2014).

While anaerobes like *P. furiosus* are potentially good producers of fermentation products, it should be remembered that they are able to extract only a fraction of the energy that aerobes obtain from the same substrates. This causes fermentative anaerobes to exist in a constant state of energy limitation, where even seemingly minor energetic costs, such as export of a final product, can be problematic (Maris et al., 2004). Therefore, expression in anaerobic hosts should be focused on pathways that are either energy-neutral or energy-yielding.

### Promise of thermophiles

It is now apparent that fossil fuels cannot continue to be used at their current rate without causing irreparable environmental harm. The shift away from petroleum will necessitate dramatic changes to the current motor-fuel regime, but will also significantly alter the production of plastics, solvents, and other specialty chemicals that are currently generated in chemical refineries. Extreme thermophiles, because of their unique advantages and the recent expansion of genetic systems allowing for metabolic engineering, are perfectly positioned to fill the need for massive chemical production from renewable feedstocks. They are able to survive the high temperatures that can result from heat generated in large-scale bioreactors, and when operated at these temperatures are less likely to be contaminated by ambient microorganisms and phages. Many also exhibit unique metabolic properties as a product of their extreme environment. Much work remains to be done before the promise of using thermophilic hosts to produce large quantities of renewable fuels and chemicals can be realized, but the genetic tools are now in place to allow that work to be carried out.

### Conflict of interest statement

The authors declare that the research was conducted in the absence of any commercial or financial relationships that could be construed as a potential conflict of interest.
